# Severe anaphylaxis caused by intravenous anti‐cancer drugs

**DOI:** 10.1002/cam4.4252

**Published:** 2021-09-10

**Authors:** Nobuyuki Horita, Etsuko Miyagi, Taichi Mizushima, Maki Hagihara, Chiaki Hata, Yuki Hattori, Narihiko Hayashi, Kuniyasu Irie, Hideyuki Ishikawa, Yusuke Kawabata, Yosuke Kitani, Noritoshi Kobayashi, Nobuaki Kobayashi, Yusuke Kurita, Yohei Miyake, Kentaro Miyake, Senri Oguri, Ichiro Ota, Ayako Shimizu, Masanobu Takeuchi, Akimitsu Yamada, Kojiro Yamamoto, Norio Yukawa, Munetaka Masuda, Nobuhiko Oridate, Yasushi Ichikawa, Takeshi Kaneko

**Affiliations:** ^1^ Chemotherapy Committee Yokohama City University Hospital Yokohama Japan; ^2^ Department of Obstetrics and Gynecology Yokohama City University Hospital Yokohama Japan; ^3^ Department of Surgery Yokohama City University Hospital Yokohama Japan; ^4^ Department of Otorhinolaryngology Head and Neck Surgery Yokohama City University Hospital Yokohama Japan; ^5^ Department of Oncology Yokohama City University Hospital Yokohama Japan; ^6^ Department of Pulmonology Yokohama City University Hospital Yokohama Japan

**Keywords:** anaphylaxis, drug hypersensitivity, medical oncology, retrospective studies

## Abstract

**Background:**

The incidence and risk factors of severe anaphylaxis by intravenous anti‐cancer drugs are unclear, whereas those of milder reactions have been reported.

**Study Design:**

Electronic medical charts of cancer patients who have undergone intravenous chemotherapy between January 2013 and October 2020 in a university hospital were retrospectively reviewed. Non‐epithelial malignancies were also included in the analysis. "Severe anaphylaxis" was judged using Brown's criteria: typical presentation of anaphylaxis and one or more of hypoxia, shock, and neurologic compromise. (UMIN000042887).

**Results:**

Among 5584 patients (2964 males [53.1%], 2620 females [46.9%], median age 66 years), 88,200 person‐day anti‐cancer drug administrations were performed intravenously, and 27 severe anaphylaxes were observed. The causative drugs included carboplatin (14 cases), paclitaxel (9 cases), and cisplatin, docetaxel, trastuzumab, and cetuximab (1 case each). The person‐based lifetime incidence of severe anaphylaxis for patients who received at least one intravenous chemotherapy was 0.48% (27/5584, 95% confidence interval (CI) 0.30%–0.67%) and the administration‐based incidence was 0.031% (27/88,200, 95% CI 0.019%–0.043%). Among 124 patients who received at least 10 carboplatin administrations, 10 patients experienced carboplatin‐induced severe anaphylaxis (10/124, 8.1%, 95% CI 3.0%–13.1%). Carboplatin caused severe anaphylaxis after at least 9‐min interval since the drip started. Thirteen out of 14 patients experienced carboplatin‐induced severe anaphylaxis within a 75‐day interval from the previous treatment. Paclitaxel infusion caused severe anaphylaxis after a median of 5 min after the first drip of the day at a life‐long incidence of 0.93% (9/968, 95% CI 0.27%–1.59%).

**Conclusion:**

We elucidated the high‐risk settings of chemotherapy‐induced severe anaphylaxis.

## Graphical abstract

88,200 person‐days of chemotherapy provided for 5584 patients were reviewed. They induced 27 severe anaphylaxes defined by Brown’s criteria. Most were caused by carboplatin (14 cases) or paclitaxel (9 cases).

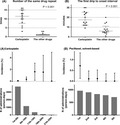

## 1 BACKGROUND

Anaphylaxis is an acute, severe, and potentially life‐threatening allergic reaction that is caused by a variety of foreign substances. Food, venom, and medications including beta‐lactam antibiotics and non‐steroidal anti‐inflammatory drugs are common triggers of anaphylaxis.^1^, ^2^ Intravenous anti‐cancer drugs such as platinum agents and taxanes also often induce hypersensitivity reactions.^3^, ^4^, ^5^ A patient with mild allergic reaction may have localized rash, itchiness, or rhinitis.^1^, ^2^ However, severe forms of drug‐induced anaphylaxis are iatrogenic critical events that demand life‐supporting maneuvers.^6^ Therefore, severe anaphylaxis should be paid careful attention. However, few studies have focused on chemotherapy‐induced severe anaphylaxis, except for case reports.^7^ Despite the recommended use of dexamethasone and H1/H2 antihistamine prophylactic pretreatment prior to chemotherapies,^5^, ^8^ some patients still experience severe anaphylaxis. Therefore, we need to know the incidence and risk factors of severe anaphylaxis caused by intravenous anti‐cancer drugs to prepare for acute‐onset critical events and to design novel regimens.

In 1999, Markman et al. studied 205 patients who were treated with carboplatin, observing that 24 (12%) of them developed hypersensitivity reactions of various severities.^8^ Their observation also demonstrated that allergic reactions mainly developed at the 8th or later carboplatin treatment.^8^ Another well‐known risk factor is carboplatin infusion after a 1‐year or longer interval.^9^, ^10^, ^11^ In contrast, based on a report by Weiss et al. in 1990, paclitaxel hypersensitivity reactions of any grade occurred in 27 of 301 patients (9%) and caused mainly by the first or second exposure to paclitaxel.^12^ However, it is not clear whether these rules can be applied for severe anaphylaxis because limited numbers of severe cases were reported in these studies. Furthermore, recently recommended prophylactic premedication might modify the epidemiology. In addition, numerous new anti‐cancer agents, especially molecular‐targeted drugs, have become available in the last two decades. Therefore, a comprehensive updated risk assessment is necessary to elucidate the incidence and high‐risk situations of chemotherapy‐induced severe anaphylaxis. The aim of this study was to assess the incidence of severe anaphylaxis caused by anti‐cancer medications and to elucidate high‐risk situations.

## 2 METHODS

### 2.1 Overview

This was a single‐center retrospective chart review study registered with the identification number UMIN000042887 and was approved by the Yokohama City University Hospital Institutional Ethical Review Board (ID: B201200069). Instead of informed consent, chance of opt out was offered for patients; however, none of the patient claimed opt out for this retrospective study.

### 2.2 Data collection

The electronic medical charts of the Yokohama City University Hospital were retrospectively reviewed. In this hospital, prophylactic medications were routinely provided prior to high‐risk agents, such as carboplatin and paclitaxel. All out‐patients and in‐patients who underwent intravenous chemotherapy for any malignancy between 1 January 2013 and 30 October 2020 were identified. Since our study focused on intravenous anti‐cancer medications, anti‐cancer drugs though other routes, such as oral, hypo‐cutaneous, or intra‐arterial administration were excluded from our analysis. Non‐epithelial malignancies such as sarcoma and leukemia were also included in our analysis.

Background data such as age, sex, type of malignancies, and chemotherapy regimens were systematically extracted. Charts of patients who were given the International Classification of Disease Code (9th or 10th edition) of anaphylaxis, anaphylactic shock, or shock on the day of the chemotherapy were scrutinized in detail.

### 2.3 Diagnosis of severe anaphylaxis

"Severe anaphylaxis" was judged using Brown's criteria,^6^ typical presentation of anaphylaxis and one or more of hypoxia with saturation of oxygen by pulse oximetry (SpO_2_) of 92% or lower, hypotension with systolic blood pressure below 90 mmHg, and neurologic compromise such as confusion, collapse, loss of consciousness, and incontinence. This criterion annotated 139 severe anaphylaxis cases (12.2%) out of 1149 patients with systemic hypersensitivity reactions due to a variety of triggers who visited or were transported to an emergency department.^6^ When an anti‐cancer drug was intravenously started and then led to an allergic reaction within 30 min, the drug was considered to be the causative agent for our analysis.^1^, ^8^ Allergic reactions to monoclonal antibodies are often termed infusion reactions as distinguished from anaphylaxis. However, as long as the clinical presentation satisfied Brown's criteria, we counted allergic reaction caused by the monoclonal antibody as severe anaphylaxis in our analysis, following recent guidelines.^13^

### 2.4 Statistics

We calculated two types of incidences in this study. For administration‐based incidence, one person‐day treatment was considered as one administration. For example, when two cycles of gemcitabine (days 1, 8, and 15) were provided to a patient, this patient is considered to have 6 day‐persons of gemcitabine treatment regardless of the cycle count. On the other hand, person‐based lifetime incidence was calculated regardless of the total number of administrations for each patient. The Agresti–Coull method was used to estimate the 95% confidence interval (CI) of incidence, and the Mann–Whitney test was used to compare nonparametric numbers in two groups.

## 3 RESULTS

### 3.1 Background data of patients and use of drugs

Between January 2013 and October 2020, 5584 patients including 2964 males (53.1%) and 2620 females (46.9%) underwent anti‐cancer chemotherapy (Table 1). The median age at the first treatment during the observation period was 66 years (range 0–93, interquartile range (IQR) 56–73, Table 1). Of the 5584 patients, the most prevalent targeted malignancy, allowing multiple‐counting for double‐ and triple‐cancer patients, was pancreatic cancer (705 patients, 11.6%, Table 1) followed by colon cancer (643, 10.6%), biliary cancer (445, 7.3%), and head and neck cancers (444, 7.3%). Patients received median of 10 anti‐cancer intravenous administrations (range 1–222, IQR 5–22).

**TABLE 1:** Background characteristics of patients

*N*	5584
Median age (interquartile range)	66 (56–73)
Man	2964 (53.1%)
Woman	2620 (46.9%)
Cancer type	
Pancreatic cancer	705 (11.6%)
Colon cancer	643 (10.6%)
Biliary cancer	445 (7.3%)
Head and neck cancer	444 (7.3%)
Breast cancer	430 (7.1%)
Non‐Hodgkin lymphoma	383 (6.3%)
Cervical cancer	333 (5.5%)
Gastric cancer	328 (5.4%)
Esophageal cancer	312 (5.1%)
Ovarian cancer	255 (4.2%)
Non‐small cell lung cancer	227 (3.7%)
Endometrial cancer	155 (2.5%)
Oral cancer	135 (2.2%)
Urothelial carcinoma	125 (2.1%)
Acute lymphatic leukemia	118 (1.9%)
Acute myelogenous leukemia	97 (1.6%)
Brain tumor	92 (1.5%)
Renal cell carcinoma	79 (1.3%)
Malignant soft tissue tumor	79 (1.3%)
Malignant melanoma	79 (1.3%)
Non‐small cell lung cancer	63 (1.0%)
Prostate cancer	58 (1.0%)

Note

Age was determined by the day of first anti‐cancer therapy during the observation period.

A patient who had double or triple cancer may be double counted in this table only for cancer type.

Cancers with prevalence >1% are listed.

A total of 88,200 person‐days of treatment were performed at our institution, of which 34,991 (39.7%) were provided in ward and 53,209 (60.3%) were provided in the Outpatient Chemotherapy Center.

The most frequently used drug, fluorouracil, was administered 14,893 times to 1066 individual patients. Drugs infused more than 2000 times such as gemcitabine, cisplatin, and solvent‐based paclitaxel are listed in Table 2.

**TABLE 2:** Frequently used anti‐cancer drugs

Drug	Administration (Person‐day)	Patients	Severe anaphylaxis
Fluorouracil	14,893	1066	0
Gemcitabine	14,575	1242	0
Cisplatin	10,305	1643	1
Paclitaxel, solvent‐based	8894	1034	9
Bevacizumab	6743	563	0
Irinotecan	6263	608	0
Levofolinate	6106	494	0
Carboplatin	5581	1037	14
Cetuximab	5239	326	1
Etoposide	4449	360	0
Oxaliplatin	4321	694	0
Paclitaxel, nanoparticle‐albumin‐bound	3692	434	0
Cyclophosphamide	3312	697	0
Vincristine	3143	483	0
Nivolumab	2927	243	0
Doxorubicin	2908	468	0
Trastuzumab, non‐conjugated	2903	166	1
Cytarabine	2676	159	0
Ifomide	2454	167	0
Mesna	2215	163	0
Rituximab	2137	319	0
Docetaxel	2079	520	1

Note

Drugs that were administrated >2000 times are listed.

nab‐Paclitaxel, nanoparticle‐albumin‐bound Paclitaxel.

### 3.2 Overall incidence

We identified 27 cases of severe anaphylaxis from our database despite 25 of them having preventive premedication, including dexamethasone (Table 3). The person‐based lifetime incidence of severe anaphylaxis for patients who received at least one intravenous chemotherapy was 0.48% (27/5584, 95% confidence interval (CI) 0.30%–0.67%) and administration‐based incidence was 0.031% (27/88,200, 95% CI 0.019%–0.043%). The causative drugs were carboplatin in 14 cases (52%), paclitaxel in 9 cases (33%), and cisplatin, docetaxel, trastuzumab, and cetuximab (1 case each, 4%) (Table 3). Notably, 19 out of 27 patients were gynecological cases (13 ovarian, 4 cervical, and 2 endometrial). Otherwise, no age‐, or sex‐related predominance observed (Tables 1, 3 and 1, 3).

**TABLE 3:** Case presentation of patients with severe anaphylaxis

Case	Prevention	Causative drug	Drug repeat	Interval	Onset (min)	Symptoms and signs	Treatment
1) 77F, Ovarian	Dex, Dph, Rnt	Carboplatin	3rd	43 d	15	Nausea, itchiness, dyspnea, generalized flush, O_2_ desaturation (87%), (relative shock (148/77 mmHg >108/75 mmHg))	O_2_, hydrocortisone
2) 49F, Ovarian	Dex, Dph, Rnt	Carboplatin	5th	75 d	14	Facial flush, itchiness, nausea, shock (87/46 mmHg), agonized look, neck discomfort, SpO_2_ 93 (2 L)	O_2_, hydration, cortisone, Fmt, CPM
3) 48F, Ovarian	Dex, Dph, Rnt	Carboplatin	6th	21 d	26	Chest distress, dyspnea, facial flush, perspiration, O_2_ desaturation (91%)	O_2_, Ad im, cortisone, Cp
4) 61F, Ovarian	Dex, Dph, Rnt	Carboplatin	8th	21 d	24	Hot flush, nausea, facial flush, perspiration, dizziness, shock (75/35 mmHg), O_2_ desaturation (89%)	hydration, Ad im, cortisone
5) 73F, Ovarian	Dex	Carboplatin	10th	24 d	30	Cervical confinement sensation, O_2_ desaturation (89%), itchiness of ear and upper arms	O_2_, hydration, Cp
6) 54F, Cervical	Dex, Dph, Rnt	Carboplatin	10th	28 d	25	Generalized flush, dyspnea, O_2_ desaturation (91%)	O_2_, hydration, cortisone,
7) 48F, Ovarian	Dex, Dph, Rnt	Carboplatin	11th	32 d	12	Discomfort, nausea, vomiting, perspiration, facial flush, dyspnea, shock (56/42 mmHg), tachycardia (135/min), O_2_ desaturation (75% on O_2_ 5 L/min mask)	O_2_, Ad im, cortisone, Cp, Fmt
8) 62F, Ovarian	Dex	Carboplatin	12th	34 d	12	Palm rash, palm itchiness, face flush, conjunctival hyperemia, faintness, shock (unmeasurable), nausea, vomiting, O_2_ desaturation (93%)	O_2_, hydration, cortisone, Cp, Fmt,
9) 70F, Ovarian	Dex	Carboplatin	13th	295 d	12	Dyspnea, O_2_ desaturation (<90%), tachypnea (23/min)	O_2_, hydration
10) 83F, Ovarian	Dex	Carboplatin	13th	28 d	9	Numb mouth, nausea, dyspnea, shock (57/35 mmHg), O_2_ desaturation (87%)	O_2_, hydration, Ad im, cortisone
11) 66F, Ovarian	Dex, Dph, Rnt	Carboplatin	15th	24 d	23	Nausea, vomiting, shock (85/64 mmHg), facial flush, arm flush, perspiration	hydration
12) 69F, Ovarian	Dex, Dph, Rnt	Carboplatin	15th	21 d	30	Palm flush, nausea, shock (55/38 mmHg), O_2_ desaturation (SpO_2_ 94), slurred speech	O_2_, hydration, cortisone
13) 54F, Cervical	Dex, Dph, Rnt	Carboplatin	16th	54 d	15	Nausea, shock (81/52 mmHg), chest compression	O3, hydration, CPM
14) 68F, Ovarian	Dex, Dph, Rnt	Carboplatin	16th	28 d	20	Nausea, facial flush, puffy lips, shock (71/50 mmHg)	O_2_, hydration, cortisone, Cp
15) 55F, Cervical	None	Cisplatin	3rd	7 d	20	Numbness, facial flush, nausea, vomiting, O_2_ desaturation (SpO_2_ < 90%), hypotension (93/51 mmHg)	O_2_, hydration, cortisone, CFM
16) 59M, Gastric	Dex, Dph, Rnt	Paclitaxel	1st	NA	15	Dyspnea, facial flush, O_2_ desaturation (85%), low‐grade fever (37.2°C), hypertension (100/77 mmHg > 177/119 mmHg)	O_2_, prednisolone, Cp
17) 68M, Esophagus	Dex, Cp, Rnt	Paclitaxel	1st	NA	4	Transient blindness, O_2_ desaturation (90% on O_2_ 4 L/min mask), shock (58/32 mmHg), bradycardia (56/min)	O_2_, hydration, Ad im, cortisone
18) 60M, Thymic	Dex, Dph, Rnt	Paclitaxel	1st	NA	3	Nausea, dyspnea, perspiration, pale face, shock (unmeasurable), unconsciousness	O_2_, hydration, Ad im, cortisone, Fmt, Cp
19) 51F, Cervical	Dex, Dph, Rnt	Paclitaxel	1st	NA	8	Fainting, shock (44/27 mmHg), abdominal pain, O_2_ desaturation (94% on O_2_ 5 L/min mask), tachycardia (128/min), hoarseness, dyspnea, rash, itchiness, incontinence	O_2_, hydration, Ad im, cortisone,
20) 46F, Uterus	Dex, Dph, Rnt	Paclitaxel	1st	NA	5	Facial flush, dyspnea, O_2_ desaturation (88%), shock (67/50 mmHg), restlessness	O_2_, hydration, Ad im, cortisone,
21) 50F, Cervical	Dex, Dph, Rnt	Paclitaxel	1st	NA	7	Dyspnea, cough, O_2_ desaturation (SpO_2_ 80%), wheeze	Ad im
22) 51F, Uterus	Dex, Dph, Rnt	Paclitaxel	1st	NA	6	Facial flush, cyanosis, O_2_ desaturation (SpO_2_ 90%)	O_2_, hydration, Fmt, CPM
23) 73F, Ovarian	Dex, Dph, Fmt	Paclitaxel	2nd	21 d	5	O_2_ desaturation (90% on O_2_ 4 L/min mask), shock (50/30 mmHg), low‐grade fever (37.1°C), generalized flush, perspiration	O_2_, Ad im x2, cortisone, saltanol inhaler, Cp, Fmt
24) 74M, Lung	Dex, Dph, Rnt	Paclitaxel	3rd	28 d	1	Unconsciousness, shock (52/40 mmHg), O_2_ desaturation (76%), perspiration	O_2_, hydration, cortisone
25) 60M, Gastric	Dex	Docetaxel	1st	NA	5	Dyspnea, O_2_ desaturation (90%), facial flush, chest discomfort	O_2_, hydration, cortisone
26) 55F, Breast	None	Trastuzumab	1st	NA	14	High fever (38.5°C), shivering, shock (unmeasurable), O_2_ desaturation (93% on O_2_ 10 L/min mask), vomiting, wheezing	O_2_, Ad im x2, Cp, benetorine nebulizer, aminophylline, Fmt
27) 71M, Head/Neck	Dex, Cp	Cetuximab	1st	NA	8	O_2_ desaturation (86%), facial flush, dyspnea	O_2_, cortisone

Note

Ad im, adrenaline intramuscular shot, cortisone: hydrocortisone. Onset, interval from the first drip of the day to the onset. Drug repeat, cumulative number of causative drug administration including the administration leading to the event. Interval, interval since the last administration of the causative drug (day).

Abbreviations: Cp, chlorpheniramine; Dex, Dexamethasone; Dph, Diphenhydramine; F, Female; Fmt, Famotidine; In‐P, Inpatient; M, Male; O_2_, oxygen; Out‐P, Outpatient; Rnt, Ranitidine.

Three severe anaphylaxes (11%) were caused by one each of brand‐name cetuximab (Erbitux), non‐conjugated trastuzumab (Herceptin), and docetaxel (Taxotere); the other 24 events (89%) were caused by generic or biosimilar medications. This only reflected the frequency of use in our hospital. Majorities of cetuximab (96.1%) and non‐conjugated trastuzumab (99.1%) and 23.7% of docetaxel were brand‐name medication, while only generic drugs were administrated for cisplatin, solvent‐based paclitaxel, and carboplatin during the study period.

### 3.3 Carboplatin and platinum

During the review period, 5581 carboplatin administrations for 1037 patients induced a total of 14 severe anaphylaxes (Table 2), which yielded an administration‐based incidence of 0.25% (95% CI 0.11%–0.39%) and person‐based lifetime incidence for patients who received at least one carboplatin administration of 1.35% (95% CI 0.60%–2.10%).

**FIGURE 1:**
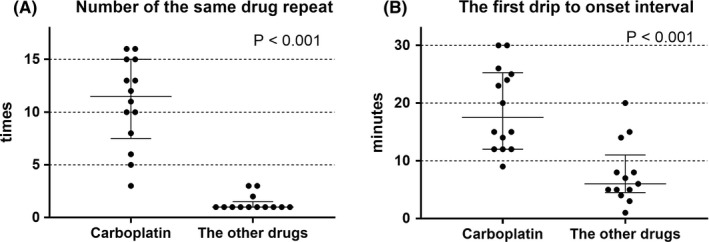
High‐risk situations of severe anaphylaxis caused by carboplatin and the other drugs. *p* value: Mann–Whitney test. Drug repeat: cumulative number of causative drug administration including the administration leading to the event. Error bar: Median and interquartile range are presented

**FIGURE 2:**
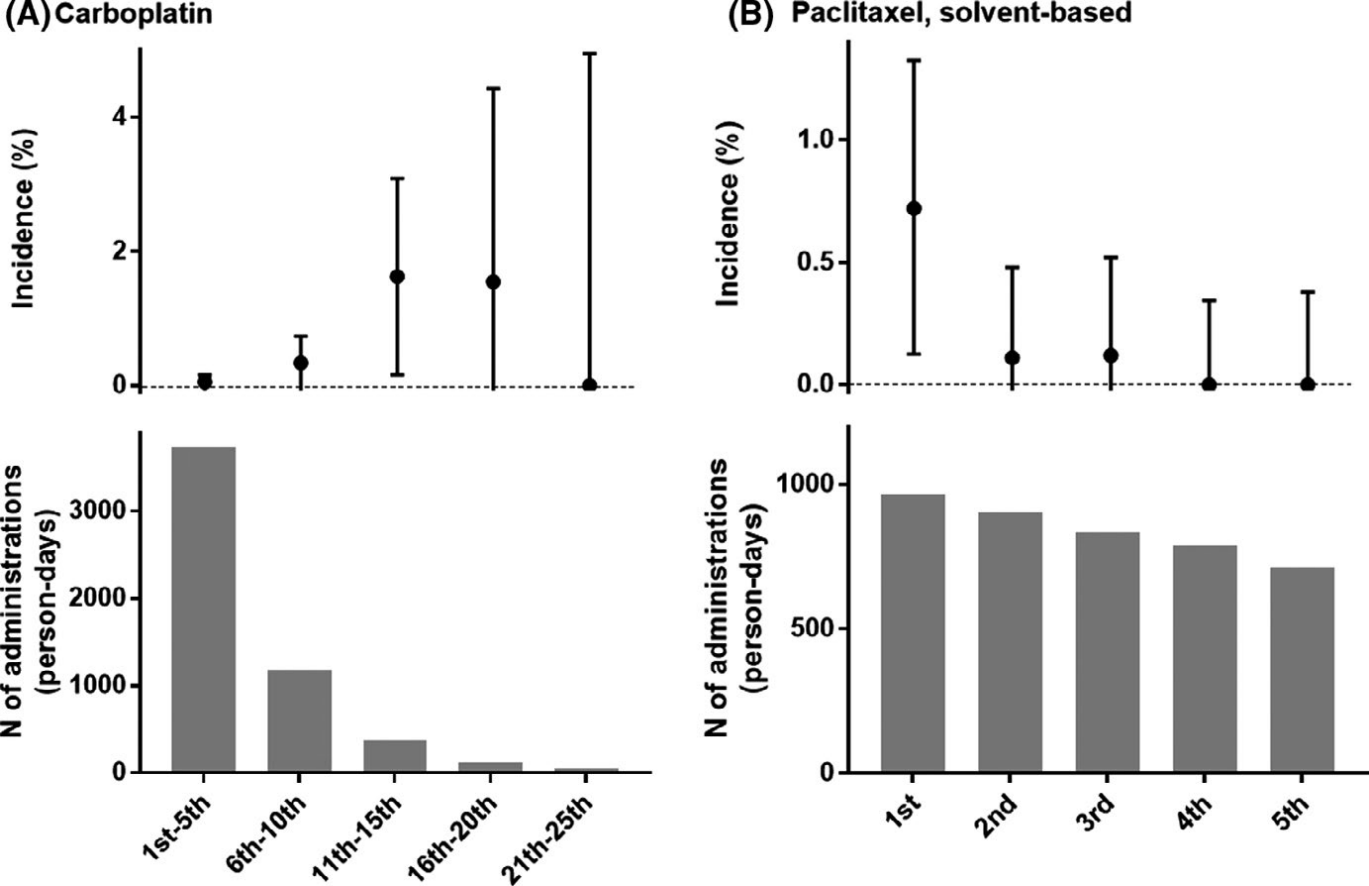
Incidence stratified by total number of causative drug administration. Error bar: Agresti–Coull method was used to estimate 95% confidence interval

According to the data of these 14 patients, the total number of carboplatin administrations including those that led to the severe anaphylaxis ranged from 3 to 16 (median 11.5) (Table 3). Compared to the 13 events caused by the other drugs, a larger number of the same drug was administered to each person until the severe anaphylaxis (*p* < 0.001, Figure 1A). Once stratified by every five cumulative carboplatin administrations, the incidence of severe anaphylaxis was highest for patients who received their 11th–15th carboplatin administration (6/369 = 1.63%, 95% CI 0.16%–3.09%) followed by the 16th–20th treatment (2/129, 1.55%, 95% CI 0%–4.43%), whereas that of the 1st–5th treatment was 0.054% (2/3740, 95% CI 0%–0.16%) (Figure 2A). Among 124 patients who received at least 10 carboplatin administrations, 10 experienced severe anaphylaxes in their life (10/124, 8.1%, 95% CI 3.0%–13.1%). Out of 369 administrations as the 11th–15th treatment of the patient, 283 (77%) were treated for ovarian, endometrial, or cervical cancers and we believe this explain why only gynecological patients experienced carboplatin‐induced events (Figure 1A).

The first sign or symptom of carboplatin‐induced anaphylaxis was observed at a median of 17.5 min (IQR 12.5–24.75) after the first carboplatin drip of the day (Table 3). Compared to the 13 events caused by the other drugs such as paclitaxel, it took longer interval from the first drip of the day to the reaction onset (*p* < 0.001, Figure 1B).

The interval from the previous carboplatin treatment was then analyzed. A median interval from the previous carboplatin administration to the administration leading to the event was 28 days. Thirteen out of 14 patients experienced carboplatin‐induced severe anaphylaxis within a 75‐day interval, whereas one patient experienced severe event at an interval of 295 days. There was no severe anaphylaxis observed after a 360‐day or longer interval (Table 3).

Cisplatin, which was administrated 10,305 times for 1643 patients (Table 2), caused one severe anaphylaxis (Table 3).

No case of severe anaphylaxes was induced by the other platinum treatments in this study, including oxaliplatin (4321 administrations, 694 patients, Table 2) and nedaplatin (505 administrations, 153 patients).

### 3.4 Paclitaxel and taxanes

Intravenous solvent‐based paclitaxel was infused 8894 times in 1034 patients (Table 2). Paclitaxel induced nine severe anaphylaxes including six cases of shock. Seven of the nine patients had severe anaphylaxis during the first administration of paclitaxel. The second and third administrations caused one severe event each (Table 3). Because 66 of the 1034 patients in our database received their first paclitaxel treatment before January 2013, the remaining 968 patients in this database received their first paclitaxel administration during our observation period. Among them, the incidence of paclitaxel‐induced severe anaphylaxis during the first administration was 0.72% (7/968, 95% CI 0.12%–1.32%) (Figure 2B). For this population, the incidence of lifelong paclitaxel anaphylaxis during the first up to the third treatment was 0.93% (9/968, 95% CI 0.27%–1.59%).

The median duration from the first paclitaxel drip of the day to the first sign or symptom of anaphylaxis was 5 min (range 1 to 15, Table 3).

Nanoparticle albumin‐bound paclitaxel (nab‐paclitaxel) was notably being increasingly administered, with 87 instances in 2013 and 718 in 2019. This drug was administered 3693 times in 434 cases, none of whom had severe anaphylaxis (Table 2).

A total of 520 patients received 2079 docetaxel infusions (Table 2). The first‐course docetaxel caused severe anaphylaxis in a 60‐year‐old gastric patient 5 min after the initiation of the infusion (Table 3).

No cases of severe anaphylaxis were caused by cabazitaxel, a recently developed taxane, which was used in 13 patients.

### 3.5 HER2 monoclonal antibody

HER2 monoclonal antibody, trastuzumab, was administrated 2903 times for 166 patients who had breast cancer, Paget's disease, or gastric cancer (Table 2). A 55‐year‐old woman with breast cancer who did not have any prophylactic premedication suffered from anaphylactic shock with desaturation 14 min after trastuzumab infusion (Table 3). Intra‐muscular adrenaline was shot within a minute since blood pressure dropped below 90 mmHg. She was recovered from shock in 2 min. She was safely retreated with trastuzumab with prophylactic dexamethasone, diphenhydramine, and ranitidine 10 days after this event and successfully underwent total 45 doses of trastuzumab. She was the only patients who received the same drug after the severe anaphylaxis in our institute.

### 3.6 EGFR monoclonal antibody

Cetuximab, an EGFR monoclonal antibody, was infused 5240 times in 249 patients with colon, oral, or head and neck cancers (Table 2). A 71‐year‐old male with head and neck carcinoma experienced a severe anaphylactic event 8 min after intravenous treatment with cetuximab (Table 3).

### 3.7 Clinical courses of severe anaphylaxis

Of the 27 patients who underwent the severe anaphylaxis, 22 (81%) had hypoxia, 15 (56%) had shock, and 6 (22%) had neurologic compromise (Table 3). In addition, 12 (44%) complained of dyspnea, 7 (26%) had noticeable perspiration, and 16 (59%) had generalized or facial flushing.

All patients were successfully treated with combinations of oxygen, hydration, intramuscular adrenaline, hydrocortisone, chlorpheniramine, or famotidine, and no one died. Intravenous aminophylline, inhaled salbutamol, and nebulized salbutamol were also used in a few patients. Intra‐muscular adrenaline was provided in 11 patients, of whom eight had active shock. Four of the eight patients who received an adrenaline shot recovered from shock within 5 min whereas each of the other four cases took 8, 12, 19 for the shock recovery, and 56 min, respectively.

## 4 DISCUSSION

Although many studies have warned about anaphylaxis caused by anti‐cancer drugs,^7^, ^8^, ^14^ the risk factors for chemotherapy‐induced severe anaphylaxis remained unclear. This is partly because most studies on this topic have been published as case repots, which are unsuitable for estimates of incidence and risk factors. By contrast, retrospective database analysis is a reasonable strategy for this clinical question given the low incidence of severe anaphylaxis. We analyzed 88,200 person‐day treatments for 5584 patients and clarified that the repeated carboplatin infusion and the first few administrations of solvent‐based paclitaxel are distinct high‐risk situations for severe anaphylaxis, despite the use of prophylactic medications. In addition to carboplatin and taxanes, numerous cytotoxic agents have been frequently used (Table 2); however, most of these agents did not cause severe events at our hospital. In a few cases, monoclonal antibodies were the causative agents.

Carboplatin is believed to induce IgE‐mediated type I hypersensitivity.^15^, ^16^ It typically occurs more than 10 min after the first drip of the 11th to 20th course of the drug (Table 3, Figures 1 and 2). This finding was compatible with numerous previous reports about carboplatin hypersensitivity of any grade.^3^, ^7^, ^8^, ^9^ However, the interval between the previous carboplatin administration and severe anaphylaxis remains controversial. O'Cearbhaill et al. reported that the median interval between prior platinum regimen and hypersensitivity reaction was 20 months.^9^ Gadducci et al. assessed 69 patients of whom nine experienced anaphylactic events and noted that carboplatin re‐treatment after an interval longer than 23.4 months was a strong risk for hypersensitivity of any severity (odds ratio 7.1, *p* = 0.013).^10^ A database analysis by Schwartz et al. involving 126 patients suggested that a prolonged platinum‐free interval longer than 24 months substantially increases severe carboplatin hypersensitivity of any severity (*p* = 0.009).^11^ Nonetheless, no patient in our hospital experienced severe anaphylaxis caused by carboplatin after a 1 year interval or longer (Table 3). By contrast, 13 out of 14 patients in our study had events within 75 days from the previous carboplatin treatment (Table 3). Although we could not detect a definite clue for this discrepancy, the high‐risk situations of severe anaphylaxis might be different from those of milder hypersensitivity. In any case, a patient with repeated carboplatin infusion should be closely monitored even after a short interval from the last treatment. Various protocols have been advocated for patients who experience carboplatin hypersensitivity.^17^, ^18^ However, re‐administration of the trigger medication that caused severe anaphylaxis cannot be justified.^4^, ^7^, ^19^ Interestingly, only gynecological patients experienced carboplatin‐induced severe anaphylaxis in our study, despite carboplatin being commonly used for a variety of cancers. This could be because 6th‐ or latter‐carboplatin administration was the risk factor for severe anaphylaxis and 6th‐ or latter‐carboplatin was mainly provided for gynecological patients (Figure 2A).

In our study, the first to third administration of taxanes caused severe anaphylaxis whereas fourth‐ or latter‐infusion did not, and most of these taxanes‐induced severe anaphylaxes occurred within 5 min. These findings consistent with those of previous studies regarding hypersensitivity of any grade.^8^, ^14^ Although some researchers mentioned that paclitaxel hypersensitivity is milder than that caused by carboplatin,^5^ we should do recognize that paclitaxel‐induced severe anaphylaxis is not rare (Table 3). Paclitaxel re‐administration may be considered once mild symptoms subside.^8^, ^20^ However, paclitaxel re‐administration after severe anaphylaxis seems too risky.^4^, ^7^, ^19^ Polyethylated castor oil solvent of paclitaxel is known to directly cause hypersensitivity via the non‐IgE mechanism.^5^, ^21^, ^22^ By contrast, nab‐paclitaxel is believed to rarely cause hypersensitivity.^5^, ^21^, ^22^ There was no nab‐paclitaxel‐induced severe anaphylaxis in our hospital, although this should be confirmed through another larger database.

Monoclonal antibodies can cause acute hypersensitivity, including anaphylaxis mediated by IgE, similar to anaphylactoid reactions, infusion reactions, and cytokine release syndrome.^23^ Cetuximab, a chimeric mouse‐human monoclonal antibody against EGFR is known to induce anaphylaxis when IgE antibodies target galactose.^23^, ^24^ A post‐marketing survey of trastuzumab, a humanized anti‐HER2 monoclonal antibody, covered 25,000 cases identified 74 patients (0.3%) with severe infusion‐related events mostly occurring shortly after infusion. Nine events leading to death were induced by the first administration of trastuzumab.^25^ Although some might consider that hypersensitivity reaction to monoclonal antibody is not anaphylaxis but an infusion reaction, two of our cases presented typical symptoms of severe anaphylaxis and had excellent reaction to adrenaline (Table 3, cases 19 and 20).^1^, ^13^

As long as medical staff are prepared for severe anaphylaxis, it is easy to diagnose because most patients present with sudden‐onset typical symptoms during intravenous treatment (Table 3). In addition, the first‐choice medical therapeutic option, intramuscular epinephrine (0.3 mg = 0.3 ml of 0.1%), is effective for most patients.^2^, ^26^ Nonetheless, experts strongly believe that epinephrine is underused despite the recommendations of guidelines.^2^, ^26^ Most clinicians are used to administering 1 mg intravenous epinephrine for cardio‐pulmonary arrested patients;^27^ however, they usually do not have enough chance to be trained administering intramuscular epinephrine for anaphylactic patients. Doctors and nurses who provide intravenous anti‐cancer drugs should know the risk situation illustrated in our analysis and be familiar with intramuscular adrenaline shots to deal with iatrogenic critical events.

The limitations of our study include the low evidence level due to the retrospective design, inconsistent prophylaxis regimens, and possibly omitted events 30 min after the first drip.

In conclusion, we conducted a retrospective database analysis of 88,200 person‐days of treatment for 5584 patients. Repeated carboplatin and the first few administrations of paclitaxel and docetaxel are high‐risk situation. The lifelong incidence of severe anaphylaxis was as high as 8.1% among patients who received at least 10 carboplatin administrations. Carboplatin typically caused severe anaphylaxis after 10‐min or longer interval since the first drip. Long interval since the last carboplatin treatment was not a risk of severe anaphylaxis. Paclitaxel‐induced severe anaphylaxis was typically caused by the first administration within 5 min from the first drip and the lifelong incidence was 0.93%. Monoclonal antibodies also caused a few severe anaphylaxes.

## CONFLICT OF INTEREST

Nobuyuki Horita reports personal fee from Taiho Pharmaceutical and research grant from Taiho Pharmaceutical and Daiichi Sankyo outside of the work. Etsuko Miyagi reports personal fee from MSD, Chugai Pharmaceutical, Takeda Pharmaceutical, and AstraZeneca and research grant from MSD and Chugai Pharmaceutical outside of the work. Taichi Mizushima reports personal fee from AstraZeneca outside of the work. Hideyuki Ishikawa reports personal fee from Novartis and Ono Pharmaceutical outside of the work. Noritoshi Kobayashi reports personal fee from Yakult Honsha and Novartis outside of the work. Nobuaki Kobayashi reports personal fee from Chugai Pharmaceutical, AstraZeneca, Boehringer Ingelheim, Ono Pharmaceutical, MSD, Bristol Myers Squibb, Eli Lilly, Kyowa Kirin and research grant from Chugai Pharmaceutical, Boehringer Ingelheim, MSD, Eli Lilly, Kyowa Kirin, Daiichi Sankyo, Pfizer outside of the work. Akimitsu Yamada reports personal fee from Chugai Pharmaceutical, Kyowa Kirin, Taiho Pharmaceutical, Daiichi Sankyo, Pfizer outside of the work. Munetaka Masuda reports research grant from Chugai Pharmaceutical, Kaken Pharmaceutical, Shionogi, Daiichi Sankyo, and Takeda Pharmaceutical outside of the work. Nobuhiko Oridate reports personal fee from Ono Pharmaceutical, Bristol Myers Squibb, Merck Biopharma, Taiho Pharmaceutical, MSD, and AstraZeneca and research grant from Ono Pharmaceutical, Merck Biopharma, and Taiho Pharmaceutical outside of the work. Yasushi Ichikawa reports personal fee from Chugai Pharmaceutical and Eli Lilly and research grant from Taiho Pharmaceutical, Chugai Pharmaceutical, Takeda Pharmaceutical, and Shionogi outside of the work. Takeshi Kaneko reports personal fee from Chugai Pharmaceutical, AstraZeneca, Bristol Myers Squibb, Eli Lilly, Taiho Pharmaceutical, Chugai Pharmaceutical, Daiichi Sankyo, Pfizer, Sanofi and research grant from MSD, Chugai Pharmaceutical, Eli Lilly, Taiho Pharmaceutical, Chugai Pharmaceutical, Daiichi Sankyo, Pfizer, Shionogi, Sanofi outside of the work.
